# Risk factors for *Streptococcus pyogenes *skin infections during an outbreak in Ethiopia: a case-control study

**DOI:** 10.1186/s12879-025-11488-z

**Published:** 2025-09-26

**Authors:** Amare Yirga Abate, Dessie Abebaw Angaw, Mesafint Fekadu Andargie, Mekuria Tilahun Kassie, Damtie Lankir Abebe

**Affiliations:** 1 Field Epidemiology resident, Department of Epidemiology and Biostatistics, Institute of Public Health, College of Medicine and Health Sciences, University of Gondar, Gondar, Ethiopia; 2 Department of Epidemiology and Biostatistics, Institute of Public Health, College of Medicine and Health Sciences, University of Gondar, Gondar, Ethiopia; 3 South Gondar Zone Health Department, Debre tabor, Ethiopia; 4 Amhara National Regional State Public Health Institute, Public Health Emergency Management Directorate, Bahir Dar, Ethiopia

**Keywords:** *S*kin lesion, Outbreak, Investigation, *Streptococcus pyogenes*, Northwest Ethiopia

## Abstract

**Background:**

Despite being in existence for hundreds of years, *Streptococcus pyogenes* remains a significant cause of global morbidity and mortality, with a particular impact in resource-limited settings like Ethiopia. Even though research on *Streptococcus pyogenes* skin infections in Ethiopia is growing, there’s a gap in identifying the potential risk factors contributing to this infection with prevention and control measures. The primary objective of this case-control study design was to identify potential *risk factors of Streptococcus pyogenes skin infections* and outbreak investigation was also undertaken to control and prevent the spread of *Streptococcus pyogenes*.

**Methods:**

A case-control study was conducted in the South Gondar Zone of Andabet and Dera districts from December 10, 2022, to January 10, 2023. An active case search was done with house-to-house by using epidemiologically linked case definitions, and a total of 914 residents were attacked by the outbreak. A face-to-face interview using a structured questionnaire was carried out to collect data. Epi Data version 4.6 and STATA version 17 software were used for data entry and analysis, respectively. Regression analysis was computed, and variables with a *P*-value of ≤ 0.05 were considered as statistically significant risk factors.

**Result:**

Group A beta-hemolytic *streptococcus pyogenes* was identified during the outbreak investigation. The attack rate of the infection was 22.2 cases per 1,000 population. The logistic regression analysis revealed that contact with cases (OR = 5.98, 95% CI: 2.91–12.25), poor personal hygiene (OR = 0.37, 95% CI: 0.2–0.66), inadequate water access for hygiene (OR = 2.2, 95% CI: 1.27–3.76), inadequate clothing practices (OR = 0.41, 95% CI: 0.23–0.70), and presence of injury (OR = 9.8, 95% CI: 5.85–18.41) were statistically significant risk factors.

**Conclusions and recommendations:**

Significant risk factors included contact with cases, poor personal hygiene, inadequate water access for hygiene and clothing practice, and injury. Improving personal hygiene, increasing water access, and injury prevention are recommended to reduce *S. pyogenes* transmission. *S. pyogenes* infection should be included in the national public health surveillance system. Longitudinal studies should be conducted to track the impacts of *S. pyogenes* infections over time in the same population.

**Supplementary Information:**

The online version contains supplementary material available at 10.1186/s12879-025-11488-z.

## Background

*Streptococcus pyogenes* is a gram-positive bacterium transmitted primarily through respiratory droplets or skin contact with broken skin that has secretions from infected sores on the skin [1]. People with underlying medical conditions, children (0 to 15 years) and the elderly, males, and pregnant and postpartum women were identified as at risk for *Streptococcus pyogenes* diseases [2]. It became a significant threat to the healthcare system, infecting more than 18.1 million people and resulting in more than 500,000 deaths each year [3, 4].

Globally, *Streptococcus pyogenes* (group A S*treptococcus)* is among the top ten leading causes of infection-related morbidity and mortality across a diverse clinical spectrum, ranging from pharyngitis and mild superficial skin infections to life-threatening systemic diseases [5–7]. Infections typically begin in the throat or skin. Mild *Streptococcus pyogenes* infections include pharyngitis and localized skin infections (impetigo), erysipelas (skin infection involving the dermis layer of the skin), and cellulitis (serious deep layers of the skin infection) [8–10].

Schools, nurseries and kindergartens, hospitals, homeless shelters, care homes, and military training facilities are the most common risk areas for *Streptococcus pyogenes* infections [4]. The risk of acquiring infection increases with inadequate housing (homelessness, household overcrowding, limited household resources, and poor housing conditions), low socioeconomic status, environmental tobacco smoke exposure, exposure to biting insects or skin injuries or diseases, poor personal and hand hygiene, and exposure to asymptomatic individuals [11, 12].

In low- and middle-income countries (LMICs), the burden of *Streptococcus pyogenes* is particularly high and the most frequent causes of skin infection [13–15]. Lack of access to clean water and sanitation facilities, contribute to the spread of skin infections in these countries. The prevalence of skin infections is significantly linked to poor and limited access to Water, Sanitation, and Hygiene (WASH) facilities and remain a major public health problem, in both international communities and Ethiopia [16, 17].

A study conducted in Gondar City, Ethiopia, showed that the overall prevalence of *Streptococcus pyogenes* in children is 11.2%, with 71.43% in urban areas and 28.57% in rural areas. Low-income parents, hospital admission, and cigarette smoking in the home were found to be substantially linked with *Streptococcus pyogenes* carriage among students [18].

From September 10, 2022, to January 10, 2023, about 914 residents were diagnosed with unspecified skin lesions from two adjacent districts of South Gondar Zone (Andabet and Dera). The primary objective of this case control study was to identify potential risk factors associated with the occurrence of a disease by comparing individuals with the disease and without the disease and to implement timely public health interventions.

## Methods and materials

### Study setting

The study was conducted in the Andabet and Dera districts of the South Gondar Zone. South Gondar Zone is one of the 22 administrative zones in the Amhara Region. Debre Tabor is the town for the zone, and it far 102 km from the regional town Bahir Dar. Andabet district is located 150 km from Bahir Dar and 91 km from Debre Tabor, and it has 26 (24 rural and 2 urban) kebeles (smallest administrative units within a districts). Dera is located 79 km from Debre Tabor and 47 km from Bahir Dar and has 39 (36 rural and 3 urban) kebeles. The total population of Andabet and Dera districts during the study period is 154,797 and 310,438, respectively. Geographically, Andabet is located at 11°10′ to 11°30′ N and 37°45′ to 38°00′ E, and Dera is located at an altitude of 11°45′ N and 37°30′ E.

### Study design and period

A case-control study design was used with a one-to-one case-control ratio from December 10, 2022 to January 10, 2023 to investigate the association between risk factor and disease condition. Individuals with the condition or disease of interest (cases) were compared to individuals without the condition (controls) to identify potential risk factors that may contribute to the presence of the disease. The cases were matched to controls which have the same geographical location with similar life style.

### Operational definitions

#### Suspected cases

A person who presented with an inflammatory process circumscribed to a region of the body, painful or not, with or without injury, edema or lumps, and/or serous or purulent secretion in the outbreak setting and period.

#### A confirmed case

A person who meets the suspected case definition and isolation of GAS by culture from the lesion swab.

#### Control

A person living in the outbreak setting neighboring the case or within the same household who may not manifest signs and symptoms of *S. pyogenes* disease for at least two weeks and had no wound lesion.

#### Epidemiologically linked cases

Suspected cases having an epidemiological link to laboratory-confirmed GAS skin infection cases.

#### Contact

A person having direct contact with the patient’s lesion or its secretion or indirectly by different inanimate objects like soap, fomites, sleeping mattresses, etc.

#### Clothing practices

A person who usually wears long trousers (trousers reaching to the foot) with shoes is considered to have adequate clothing practices, and a person who wears short trousers (trousers reaching to the knee) without shoes is considered to have inadequate clothing practices.

#### Inadequate water access for hygiene

A person who got less than 15 to 20 L of water per individual daily.

#### Personal hygiene

A person who washes his body greater than the high frequency of body washing (4–6 times per week) is considered to have good personal hygiene, and a person who washes his body below the less frequency of body washing per week (2–3 times per week) is considered to have poor personal hygiene [16].

### Data collection process and instrument

To collect socio-demographic characteristics and identify risk factors for *Streptococcus pyogenes* skin infections, we conducted interviews with both cases and controls using pre-tested structured questionnaires developed from various sources. The data collection process was started after ethical approval and tool development. Data collection timelines included recruitment of cases, interviews, tracking of daily collected data, data cleaning and exporting, an active case search through house-to-house visits in all affected kebeles, daily case documentation, and tracking of daily cases throughout the study period.

### Inclusion and exclusion criteria

Individuals who met the definition of cases and controls were included as cases and controls in the study, respectively. Individuals who had unspecified skin lesions and a lesion healed during the data collection period were excluded from the study.

### Sample size and sampling technique

The total sample size for this study was 652 (326 cases and 326 controls in the ratio of one to one). To compare the likelihood of an outcome (infection) between the exposed and unexposed groups and to adjust differences in proportion by incorporating the odds ratio, we used the Fleiss formula for determining sample size using the Epi Info Version 7.2.1.0 software by considering one variable assumed to bring a difference in the cases and control with a two-sided CI = 95% and Power = 80%. Inadequate water access for hygiene was considered the main predictor of the outcome, with control exposed at 10% and an OR of 2.04 [19]. Cases were selected using a simple random sampling technique from the outbreak registration tool (line listing of cases) up to the recommended sample size. One control was selected for each enrolled case within the same families or from the neighboring houses by a simple random sampling technique from the Kebele household family registration book.

### Enrolment of cases and controls

Confirmed and epidemiologically linked *Streptococcus pyogenes* skin infection cases were considered for the study. A case was defined as showing signs and symptoms of *Streptococcus pyogenes* skin infection greater than or equal to two weeks with at least one of the following signs or symptoms: lesion, itching, pain, headache, fever, or lymph node swelling between December 10, 2022 and January 10, 2023. A control was defined as any person without any signs and symptoms of *S. pyogenes* skin infection during the outbreak investigation from December 10, 2022, to January 10, 2023, in the Andabet and Dera districts within the study period.

### Specimen collection, transportation, and microbial isolation

A wound swab sample was collected from suspected cases by a sterile wooden swab with a test tube and transported without an ice pack to the bacteriology reference laboratory in the Amhara National Regional State Public Health Institute for the isolation of the causative agent. The collected sample was inoculated on a blood agar plate and MacConkey agar.

### Data quality control

Members of the team were discussed on the questionnaires in detail to have a common understanding of the tool. The collected data was cross-checked daily through recorded cases before and during data entry and processing for its completeness and consistency in the study period.

### Variables

#### Dependent variable

Participant status (case or control).

#### Independent variables

Socio-demographic characteristics (age, sex, family size, educational status, occupation, marital status, place of residence), factors impairing the skin’s intactness (trauma to the skin, other skin infections like scabies, eczema, myositis, chronic swelling of the legs or arms, obesity, immunosuppressant diseases such as diabetes, HIV, malnutrition, and the presence of chronic disease), and behavioral factors (contact with the patient, family member with a lesion, smoking status, alcoholism, clothing practices, source of water for hygiene, and personal hygiene).

### Data processing and analysis

The data were cross-checked daily through line-listed cases for their completeness and consistency. The collected data was entered into Epi Data version 4.6. STATA version 17 was used for analysis. Descriptive statistics were used to present socio-demographic variables. Binary logistic regression analysis was done, and all variables that were found to be significant at *p*-value < 0.25 were entered into the multivariable logistic regression analysis using the backward LR method. The Hosmer-Lemeshow goodness-of-fit test was used to test the model fitness, which was > 0.05. Explanatory variables were tested for multicollinearity before entering them into the multivariable model, using the variance inflation factor (VIF). Independent variables that have a *p*-value of ≤ 0.05 in the multivariable logistic regression analysis were considered significant factors for *S. pyogenes* skin infection. Finally, frequencies, figures, and tables were used to summarize the findings of the study.

### Ethical considerations

Ethical approval was obtained from the South Gondar Zone Health Department Ethical Review Committee with issue number and date as SGZ/14/435/15, 12/04/2015. The purposes and the importance of the study were stated to the Andabet and Dera districts health offices and to the cluster health centers for their cooperation and facilitating the outbreak investigation. The purposes of the outbreak investigation were explained to respondents, and written informed consent was obtained from all participants and the parent or guardian for those under 16 years old and for illiterate participants before interviewing and agreeing to take part in the investigation. Confidentiality was assured at all levels of the study using password-protected computers and through deleting all identifiers. Participants were fully autonomous for their participation. Conflicts of interest were disclosed. Force or pressure for participation was avoided. Independent health professionals were assigned to protect the life, privacy, and dignity of the participants. All vulnerable groups and individuals specifically received protection.

## Result

### Distribution of cases by place and person

A total of 914 cases were reported from the Andabet (775 cases) and Dera (139 cases) districts in the South Gondar Zone. The highest attack rate was observed in the Mekane Selassie Kebele with 96.9 cases per 1,000 population, while the lowest attack rate was found in the Dasqua Kebele with 0.7 cases per 1,000 population. Overall, the attack rate in the affected kebeles was 22.2 cases per 1,000 population (Table 1). Deaths were not reported in any of the outbreak-affected kebeles in the districts.

**Table 1:** Attack rate of *S. pyogenes* skin infection in the Andabet and Dera districts of South Gondar zone, Northwest Ethiopia, 2023

S. No	Districts	Kebeles	Total population of affected kebeles	Cases			Attack rate per 1,000 population
				Male	Female	Total	
1	Andabet	Mekane Selassie	4749	311	149	460	96.9
2	Andabet	Met kolla	4457	187	92	279	62.6
3	Andabet	Afenkir	3604	24	12	36	10.0
4	Dera	Afay	6042	66	28	94	15.6
5	Dera	Micho	8766	19	7	26	3.0
6	Dera	Deguat	6391	9	5	14	2.2
7	Dera	Dasqua	7118	4	1	5	0.7
Total			41,127	620	294	914	22.2

*NB* Kebeles are the smallest administrative units within a districts

**Fig. 1:**
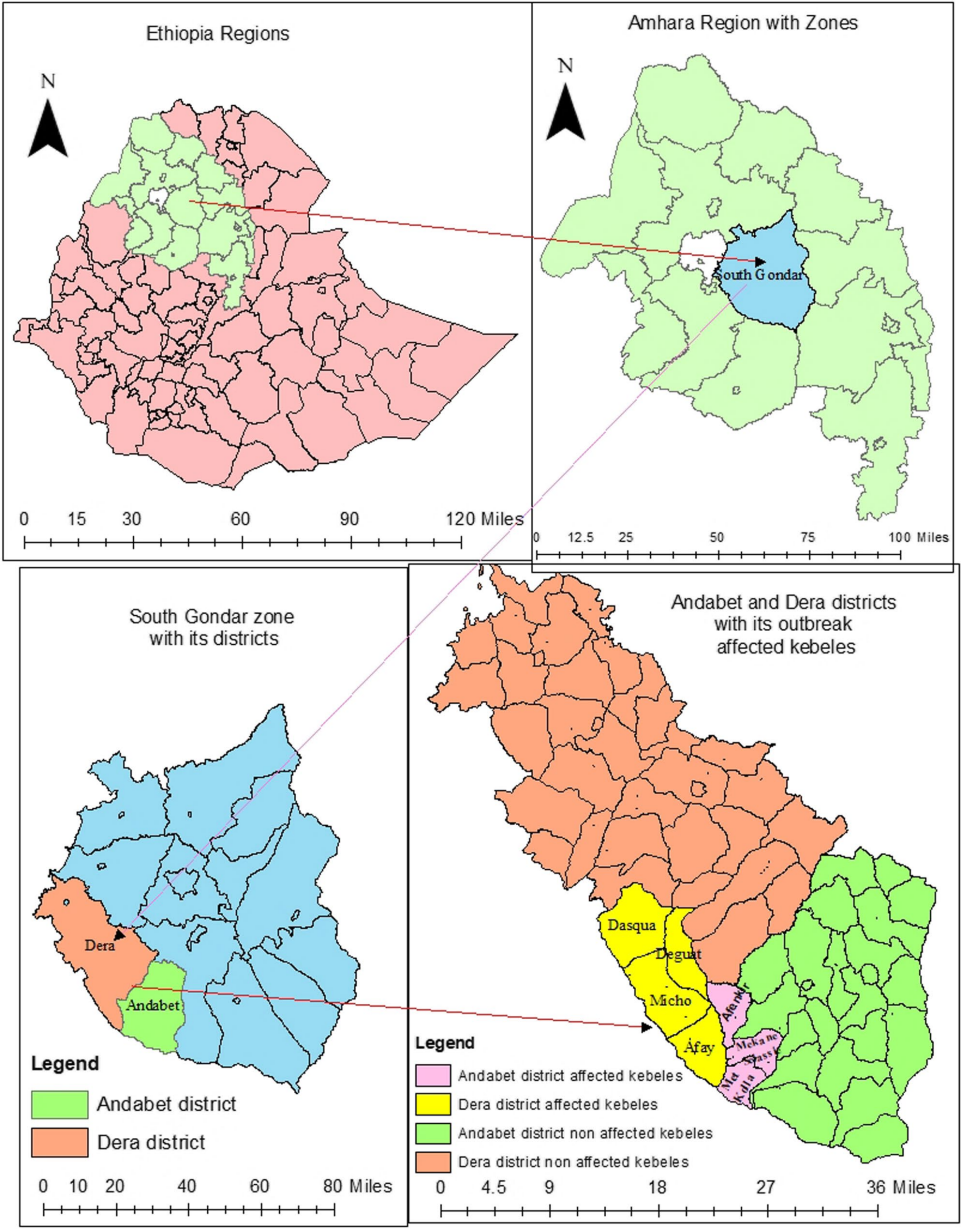
Map of Dera and Andabet districts with its *Streptococcus pyogenes* affected kebeles in South Gondar Zone. Source:- Arc GIS Version 10.8.12790 Environmental System Research Institute, esri 2019, Ethiopia Admin boundaries

*Streptococcus pyogenes infections* were widely distributed in seven kebeles (four kebeles from the Dera district and three kebeles from the Andabet district) of the South Gondar Zone. A map of the Dera and Andabet districts with their *Streptococcus pyogenes-*affected kebeles is presented in Fig. 1.

### Clinical features of cases

The predominance of lower extremity lesions from all cases was 765 (83.7%). Seven hundred and forty (81%) cases had a serosanguineous wound (blood serum secretion). Severe pain in the wound area was reported in 858 (94%) cases. Fever was not reported from 866 (94.7%) of cases (Table 2).

**Table 2:** Clinical features of *S. pyogenes* cases in Andabet and Dera districts of South Gondar zone, Northwest Ethiopia, 2023

Attributes	Response category	Male	Female	Frequency	%
Characteristic of the lesion	Purulent discharge	95	42	137	15.0
	Serosanguineous secretion	503	237	740	81.0
	dry with crust	22	15	37	4.0
Site of the lesion	Neck and above	19	9	28	3.1
	Truck	3	0	3	0.3
	upper extremity	71	47	118	12.9
	lower extremity	506	259	765	83.7
Fever	Yes	27	21	48	5.3
	No	593	273	866	94.7
Headache	Yes	217	61	278	30.4
	No	403	233	636	69.6
Sever pain	Yes	589	269	858	93.9
	No	31	25	56	6.1
Itching	Yes	22	14	36	3.9
	No	598	280	878	96.1
Lymph node Swelling	Yes	267	123	390	42.7
	No	353	171	524	57.3
Other (GI symptom, cough)	Yes	25	23	48	5.3
	No	595	271	866	94.7

### Distribution of cases by date of onset of disease

**Fig. 2:**
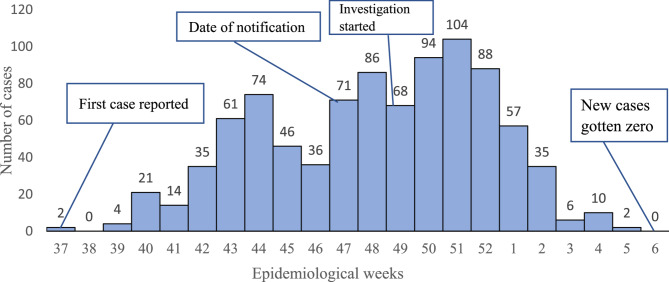
Epidemic curve of the *S. pyogenes* skin infection outbreak in Andabet and Dera district of South Gondar Zone, Northwest Ethiopia, 2023

The epidemic showed a propagated pattern, beginning with a single case and gradually increasing after two weeks and showed upslope curve. The duration of the epidemic covered 21 weeks from (Epidemiological (EPI)) week 37 to 52, 2022, and EPI week one to five, 2023, of *the S. pyogenes* outbreak that occurred in the Andabet and Dera districts of the South Gondar Zone. The first date of onset of disease reported by the respondent was September 10, 2022 (EPI week 37), and the investigation was started on EPI week 49 due to late notification of cases to the districts. The outbreak was controlled after nine weeks of investigation, and newly reported cases have been zero after EPI week six, 2023 (Fig. 2).

### Laboratory confirmation and verification of the diagnosis

To identify the causes of the infection, eleven wound swabs from the lesions of eleven individuals were collected. To differentiate bacterial characteristics, the collected samples were inoculated to blood agar plates (BAP) and MacConkey agar. Growth was seen only in BAP. Gram’s stain was done, and gram-positive cocci were identified by microscopic examination. After growth identification, a biochemical test was done, and the test result showed negative for catalase and positive for beta hemolytic. *Streptococcus pyogenes* (group A beta-hemolytic *streptococcus*) was isolated from the six samples, and *Staphylococcus aureus* was found in two wound swabs. To differentiate possible resistance and susceptible drugs for the pathogen, the bacitracin sensitivity test was placed on a blood agar plate inoculated with the *S. pyogenes* colonies. After incubation, a zone of inhibition (area where bacterial growth is absent around the disk) was observed, and all samples were sensitive to the bacitracin test. All identified pathogens are 100% susceptible to penicillin and ampicillin.

### Multivariable regression risk factors analysis

A total of 652 participants with a one-to-one case-control ratio were included in this study for risk factor analysis. From the total study subjects, 90 (27.0%) cases and 20 (6.1%) controls had contacts with cases. A total of 226 (69.3%) cases and 110 (33.7%) controls had a shortage of water for personal hygiene. A total of 208 (63.8%) cases and 74 (22.7%) controls had a wound lesion in the family members (Table 3).

**Table 3:** Bivariable and multivariable logistic regression analysis of risk factor for *S. pyogenes* skin infection in Andabet and Dera districts of South Gondar zone, Northwest Ethiopia, 2023

Variables	Participants status		COR (95% CI)	*P*. Value	AOR (95% CI)	*P*. Value
	Cases *N* (%)	Controls *N* %)				
Sex						
Male	209 (64.1)	205 (62.9)	1.05 (0.77–1.45)	0.17	1.27 (0.89–2.1)	0.09
Female	117 (35.9)	121 (37.1)	1		1	
Occupation						
Farmer	193 (59.2)	182 (55.8)	1.36 (1.05–5.51)	0.31		
House wife	47 (14.4)	64 (19.6)	0.94 (0.32–19.03)	0.69		
Student	79 (24.2)	71 (21.8)	1.43 (0.45–42.90)	0.48		
Others	7 (2.15)	9 (2.76)	1			
Education level						
Can’t read and write	212 (65.0)	188 (57.7)	0.64 (0.32–7.42)	0.29		
Can read and write	16 (4.9)	41 (12.6)	0.22 (0.11–48.27)	0.55		
Grade 1 to 8	72 (22.1)	61 (18.7)	0.67 (0.36–2.46)	0.28		
Grade 9 to 12	19 (5.83)	32 (9.8)	0.33 (0.17–3.86)	0.36		
Diploma and above	7 (2.15)	4 (1.2)	1			
Marital status						
Single	196 (60.1)	155 (47.5)	1		1	
Married	122 (37.4)	167 (51.2)	0.58 (0.17- 2,82)	0.17^*^	0.3 (0.09–4.23)	0.79
Widowed	2 (0.6)	3 (0.9)	0.52 (0.027–4.86)	0.72	0.31(0.04–4.86	0.21
Divorced	6 (1.8)	1 (0.3)	4.7 (0.66–21.24)	0.12	0.11(0.02–2.7)	0.09
Family size						
≤ 5	181(55.5)	144 (44.2)	1.6 (1.02-5,71)	0.08^*^	1.05 (0.89–2.44)	0.07
>5	145 (44.5)	182 (55.8)	1		1	
Family member with lesion						
Yes	208 (63.8)	74 (22.7)	6.00 (3.07–9.13)	< 0.001^*^	4.3 (2.65–6.95)	< 0.001**
No	118 (36.2)	252 (77.3)	1		1	
Contact with case						
Yes	90 (27.0)	20 (6.1)	5.83 (2.91–12.25)	< 0.001^*^	5.9 (2.91–12.3)	< 0.001**
No	236 (72.4)	306 (93.9)	1		1	
Inadequate water access for hygiene						
Yes	226 (69.3)	110 (33.7)	4.44 (1.27–7.76)	0.02^*^	2.2 (1.27–3.76)	0.013**
No	100 (30.7)	216 (66.3)	1		1	
Personal hygiene						
Good	174 (53.4)	63 (19.3)	1		1	
Poor	152 (46.6)	263 (80.7)	0.2 (0.12–0.83)	< 0.001^*^	0.37 (0.2–0.66)	< 0.001**
Source of water for hygiene						
Pipe	1 (0.3)	2 (0.6)	1		1	
Well	176 (54.0)	135 (41.4)	1.86 (1.14–3.02)	0.08^*^	1.05 (0.8–3.02)	0.23
River	149 (45.7)	189 (58)	2.01 (1.57–6.11)	0.14^*^	1.9 (0.85–3.71)	0.19
Presence of chronic disease						
Yes	18 (5.5)	4 (1.2)	4.7 (1.9-21.23)	0.09^*^	7.7 (1.75–33.58)	0.012**
No	308 (94.5)	322 (98.8)	1		1	
Clothing practices						
Adequate	249 (76.4)	204 (62.6)	1		1	
Inadequate	77 (23.6)	122 (37.4)	0.52 (0.35–0.75)	< 0.001*	0.41(0.23–0.70)	< 0.001**
History of injury						
Yes	209 (64.1)	40 (12.3)	12.79 (8.1–21.2)	< 0.001^*^	9.8 (5.85–18.4)	< 0.001**
No	117 (35.9)	286 (87.7)	1		1	

*NB*

* Nominated variables for multiple logistic regression analysis and *P*- Value with

** showed statistically significant at 0.05

“Others” represents for merchants and government employee

“1” represents reference value

Variables with a *P*-value ≤ 0.25 in the bivariate logistic regression were taken to the multivariable logistic analysis, and adjusted odds ratios were computed to control the effect of confounding. In multivariable logistic regression analysis, a person having contact with cases is 5.9 times more at risk to develop *S. pyogenes* skin infection than having no contact with cases (OR = 5.9, 95% CI: 2.91–12.3). The presence of chronic disease is 7.7 times more likely to develop the disease as compared to having no chronic health problem (OR = 7.7, 95% CI: 1.75–33.58) (Table 3).

### Intervention measure taken

Case management was done according to the laboratory result and all cases were managed with antibiotics. Wound care was given to 171 cases during the home-to-home new case search. All cases are linked to the cluster health center for further wound care until the wound heals. Health education was given to the residents of outbreak-affected kebeles at school, church, and community gathering places related to the mode of transmission and way of prevention for *S. pyogenes* skin infection. Key messages were communicated to individuals who have any lesions: they should contact health professionals immediately, avoid direct contact with cases, and keep up their own personal hygiene.

## Discussion

The overall attack rate of *Streptococcus pyogenes* was 22.2 cases per 1,000 population, which was higher than the epidemics of necrotizing fasciitis in the Democratic Republic of Sao Tome and Principe (15.5 cases per 1,000 population) [20]. The community’s lifestyle may be the cause of the variation, and lack of hygiene in the research area may make the illness more contagious.

This study results showed that a person who has a contact with cases has six times more risk to develop *S. pyogenes* infection as compared to a person who does not have a contact with case (OR = 5.9, 95% CI: 2.91–12.3). This study finding is similar to a review conducted in Auckland, New Zealand [21] and Australia [4]. The similarity of the finding may be due to low-income communities being heavily burdened by *S. pyogenes* infection because of their low socioeconomic status, poor housing conditions, inability to afford health care access, and other factors.

Improper hygiene practices were associated with skin infection in this study. Individuals with good personal hygiene are 63% less at risk of developing *S. pyogenes* skin infection than those who have poor personal hygiene (OR = 0.37, 95% CI: 0.2–0.66). This study result is similar to a study conducted in Mekelle city, Northern Ethiopia [22]. This study results also showed that a person who has adequate clothing practices is 59% less at risk to develop skin infection as compared to a person who has inadequate clothing practices (OR = 0.41, 95% CI: 0.23–0.70). The lower risk of developing *S. pyogenes* skin infections among individuals with good personal hygiene and adequate clothing practices in Ethiopia may be attributed to a combination of environmental, socioeconomic, cultural, and healthcare access factors.

In this study, a person having a known chronic health problem has eight times more at risk to develop *S. pyogenes* skin infection as compared to a person who has no chronic health problem (OR = 7.67, 95% CI: 1.75–33.58). The finding might be due to the fact that handling mechanism of chronic health problems, follow-up of chronic diseases, and the type of drug they used for chronic health problem management may increase the risk of *S. Pyogene* skin infection in the study area.

According to the findings of this study, individuals with a history of injury have nine times the risk of developing *S. pyogenes* skin infection compared to those without an injury (OR = 9.8, 95% CI: 5.85–18.4). This study finding is similar to the study result conducted in the Ontario, Canada [12]. This similarity might be due to the fact that broken and damaged skin is comfortable for the multiplication and growth of *S. pyogenes* skin infections easily.

In this study, family size and gender are not significantly associated with *S. pyogenes* skin infections. But the incidence of *S. pyogenes* skin infection in men is 620 (67.8%) compared with females. Higher *S. pyogenes* skin infection in males is reported with a review conducted in Auckland, New Zealand [21]. Men may be less consistent with hygiene practices; behavioral and occupational factors of men working in open environments will increase the risk of infection. This finding should require further investigation.

This study’s results showed that, a person who has inadequate water access for hygiene is two times more risk to develop *S. pyogenes* infection as compared to a person having adequate water access for hygiene (OR = 2.2, 95% CI: 1.27–3.76). Water access is critical to ensuring a healthy lifestyle. The association of risk of S. pyogenes infection and inadequate water access for hygiene may be due to many people and communities in different parts of Ethiopia region is still lacking access to safe water and improved sanitation facilities [23]. Rural areas are the most affected by barriers to improved water access for hygiene as compared to urban settings.

### Limitation of the study

The source of infection for the index case was not identified, which hinders risk assessment. The date of case notification to the zone health department was delayed, which might affect outbreak characterization with underestimate the attack rate of the infection, presence of inaccurate epidemic curve, reduced effectiveness of prevention measures, misidentification and determining the outbreak’s source and spread. Age groups of study participants were not available, and age-specific attack rates were not calculated. Some of the events may be difficult to remember, and hence the effect of recall bias may exist in related to self-reported hygiene practices and contact to cases.

## Conclusions and recommendations

The overall attack rate of the outbreak was 22.2 cases per 1,000 population in the affected districts. Contact with cases, poor personal hygiene, inadequate water access for hygiene, inadequate clothing, and presence of injury are significant risk factors for *S. pyogenes* skin infection. Improving water access, sanitation and personal hygiene and injury prevention can significantly reduce the burden of skin diseases and improve overall public health. To prevent progression and complications from *S. pyogenes* infection, regular use of closed protective footwear and early detection & treatment should be practiced. *S. pyogenes* infection should be included in the national public health surveillance system for timely response. Longitudinal studies should be conducted to track impacts of *S. pyogenes* infections over time in the same population and to address potential influence of environmental factors and access to healthcare.

## Supplementary Information

Supplementary Material 1.

Supplementary Material 2.

## Data Availability

The authors confirm that all data underlying the findings are fully available without restriction. All relevant data are within the manuscript.Clinical trial number: not applicable.

## Abbreviations

APHI: Amhara public health institute
AOR: Adjusted odds ratio
AR: Attack rate
BAP: Blood agar plates
CI: Confidence interval
COR: Crude odd ratio
EPI: Epidemiological week
GAS: Group A beta hemolytic streptococcus
LMICs: Low- and middle-income countries
PCR: Polymerase chain reaction
PHCU: Primary health care unit
PHEM: Public health emergency management
WASH: Water, sanitation, and hygiene
