# Supplementary material for: Risk factors for Streptococcus pyogenes skin infections during an outbreak in Ethiopia: a case-control study
Source: BMC Infect Dis. 2025 Sep 26;25:1150. doi: 10.1186/s12879-025-11488-z (PMC12465534; doi:10.1186/s12879-025-11488-z)
Supplement: Supplementary file 1 — Supplementary Material 1. [file 12879_2025_11488_MOESM1_ESM.docx]

Questionnaire

Data collection tool for Case - control study of unspecified skin lesion outbreak, Andabiet and Dera District, South Gondar Zone, Amhara Region, Ethiopia, 2022

Participant Code____________________________, Date of data collection___________ Region________ Zone_______ Woreda______ Kebele _______ Got _______

Participant statues case control

1. Socio-demographic Characteristics

| S. N | Question | Response | If no Skip to question No |
| --- | --- | --- | --- |
| 101 | Sex | 1. Male 2. Female |  |
| 102 | Age in year |  |  |
| 103 | Occupation | 1. Farmer 2. Housewife 3. Student 4. Merchant 5. Gov’t Employee 6. Other (specify) |  |
| 104 | Religion | 1. Orthodox 2. Muslim 3. Protestant 4. Catholic 5. Other (specify) |  |
| 105 | Educational level | 1. Can not read and write 2. Can read and write 3. Garde 1 to 8 4. Grade 9 to 12 5. Diploma and above |  |
| 106 | Marital status | 1. Single 2. Married 3. Widowed 4. Divorced 5. Other (specify) |  |
| 107 | Number of individuals living in your home (Family size) | 1. ≤ five 2. > five |  |

II clinical feature (only for cases)

| 201 | Date onset of the skin lesion (DD/MM/YY) | __________________ |  |
| --- | --- | --- | --- |
| 202 | Characteristic of the lesion | 1. Purulent discharge 2. Serosanguinous secretion 3. dry with crust |  |
| 203 | Site of the lesion | 1. Neck and above 2. Truck 3. Upper extremity 4. Lower extremity 5. Others ( specify) |  |
| 204 | Do you have any of the following sign and symptom? |  |  |
|  | Fever | 1. Yes 2. No |  |
|  | Headache | 1. Yes 2. No |  |
|  | Sever pain | 1. Yes 2. No |  |
|  | Itching | 1. Yes 2. No |  |
|  | Lymph node Swelling | 1. Yes 2. No |  |
|  | Other (GI symptom, cough) | 1. Yes 2. No |  |
| 205 | Do you visit health facility for this case? | 1. Yes 2. No |  |
| 206 | If yes, date of visit (DD/MM/YY) | __________________ |  |

III Risk factors

| 301 | Is their family member with lesion from the wound? | 1. Yes 2. No |  |
| --- | --- | --- | --- |
| 302 | Have you a contact history with lesion from the wound in the family members? | 1. Yes 2. No | If No pass to 304 |
| 303 | If yes, date of contact (DD/MM/YY) |  |  |
| 304 | Is there a shortage of water for hygiene? | 1. Yes (got less than 15 to 20 litter of water per individual daily) 2. No (got greater than 15 to 20 litter of water per individual daily) |  |
| 305 | Personnel hygiene | 1. Good (washes greater than once in a week) 2. Poor (washes less than once in a week) |  |
| 306 | What is the source of water for hygiene? | 1. Pipe 2. Well 3. River 4. Other (specify) |  |
| 307 | Do you have a known chronic disease? | 1. Yes 2. No | If No pass to question 309 |
| 308 | If yes, what type of chronic diseases do you have? | 1. Diabetes Mellitus 2. Cardiovascular disease 3. Kidney disease 4. Hypertension 5. Other (specify) |  |
| 309 | Wearing style | 1. Good (Wears long trouser) 2. Poor (Wears short trouser) |  |
| 310 | Have you a history of injury | 1. Yes 2. No |  |
