# Supplementary material for: Risk factors for Streptococcus pyogenes skin infections during an outbreak in Ethiopia: a case-control study
Source: BMC Infect Dis. 2025 Sep 26;25:1150. doi: 10.1186/s12879-025-11488-z (PMC12465534; doi:10.1186/s12879-025-11488-z)

Maps of Dera and Andabet districts with its *Streptococcus pyogenes* affected kebeles in South Gondar Zone.

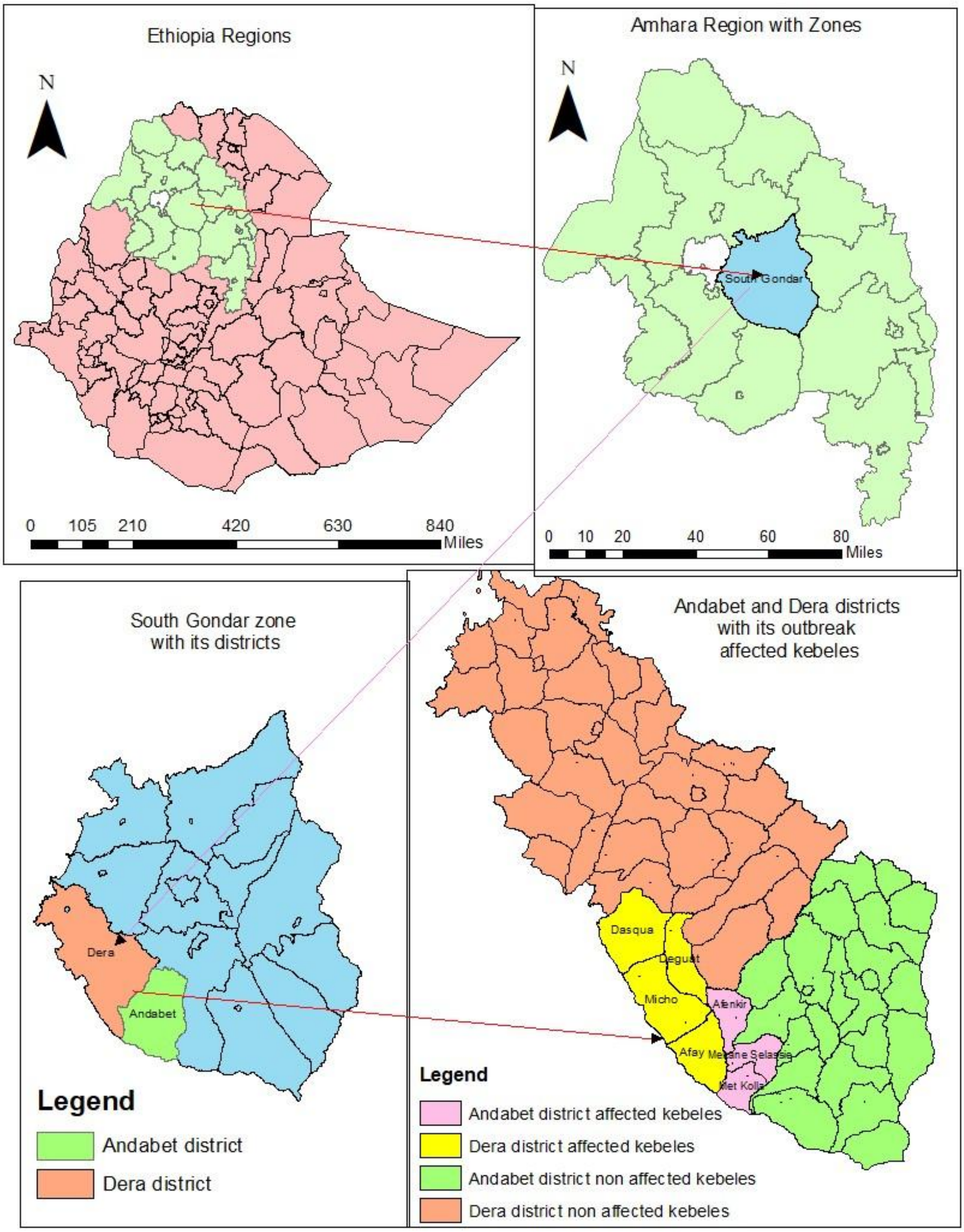

Supplement: Supplementary file 2 — Supplementary Material 2. [file 12879_2025_11488_MOESM2_ESM.pdf]
